# A rare case of anomalous origin of the left main coronary artery in an adult patient

**DOI:** 10.1186/1749-8090-8-15

**Published:** 2013-01-22

**Authors:** Pierre O Dionne, Nancy Poirier, Jessica Forcillo, Louis M Stevens, Carl Chartrand-Lefebvre, Samer Mansour, Nicolas Noiseux

**Affiliations:** 1 Division of cardiac surgery, Centre Hospitalier de l’Université de Montréal (CHUM), St Urbain Street, Montréal, H2W 1T8, Canada; 2 Division of cardiac surgery, Centre Hospitalier Universitaire Mère-Enfant (CHU-ME), Chemin de la Côte-Sainte-Catherine, Montréal, H3T 1C5, Canada; 3 Division of cardiac surgery, Centre Hospitalier de l'Université de Montréal (CHUM) et Centre de recherche du CHUM (CRCHUM), St Urbain Street, Montréal, H2W 1T8, Canada; 4 Department of radiology, Centre Hospitalier de l'Université de Montréal (CHUM) et Centre de recherche du CHUM (CRCHUM), St Urbain Street, Montréal, H2W 1T8, Canada; 5 Division of cardiology, Centre Hospitalier de l'Université de Montréal (CHUM) et Centre de recherche du CHUM (CRCHUM), St Urbain Street, Montréal, H2W 1T8, Canada

**Keywords:** Congenital heart defect, Cardiac surgery, Coronary artery bypass graft

## Abstract

Anomalous origin of left coronary artery from the pulmonary artery (ALCAPA) is a rare congenital anomaly that causes a left-to-right shunt via the coronary system, resulting in coronary steal. We report an unusual case of a healthy 48 years-old patient presenting with dyspnea on exertion and mild chest pain who underwent surgical correction of this rare anomaly. Multiple procedures have been proposed in adults with ALCAPA. Although re-implantation of the left main coronary artery (LMCA) to the aorta remains the most physiological correction for this anomaly, the combination of LMCA ligation and coronary artery bypass grafting provides a dual coronary flow system and is preferable when re-implantation is impossible.

## Background

Anomalous origin of left coronary artery from the pulmonary artery (ALCAPA) is an uncommon congenital anomaly affecting 1/300,000 live births [1]. As pulmonary artery (PA) pressure lowers to a third that of systemic pressure during the neonatal period, left coronary flow will tend to reverse into the pulmonary artery (PA) resulting in coronary steal and a left to right shunt, with subsequent decreased myocardial perfusion and volume overload. The vast majority of patients present with heart failure secondary to myocardial ischemia or die during infancy. Because patients with symptoms should be operated, it is seldom identified in adults. Survivors have developed significant collateral flow from the right coronary artery (RCA) and secondary dilatation of the coronary arteries.

We present herein an unusual case of ALCAPA seen in one of the oldest patient ever reported.

## Case presentation

**Figure 1:**
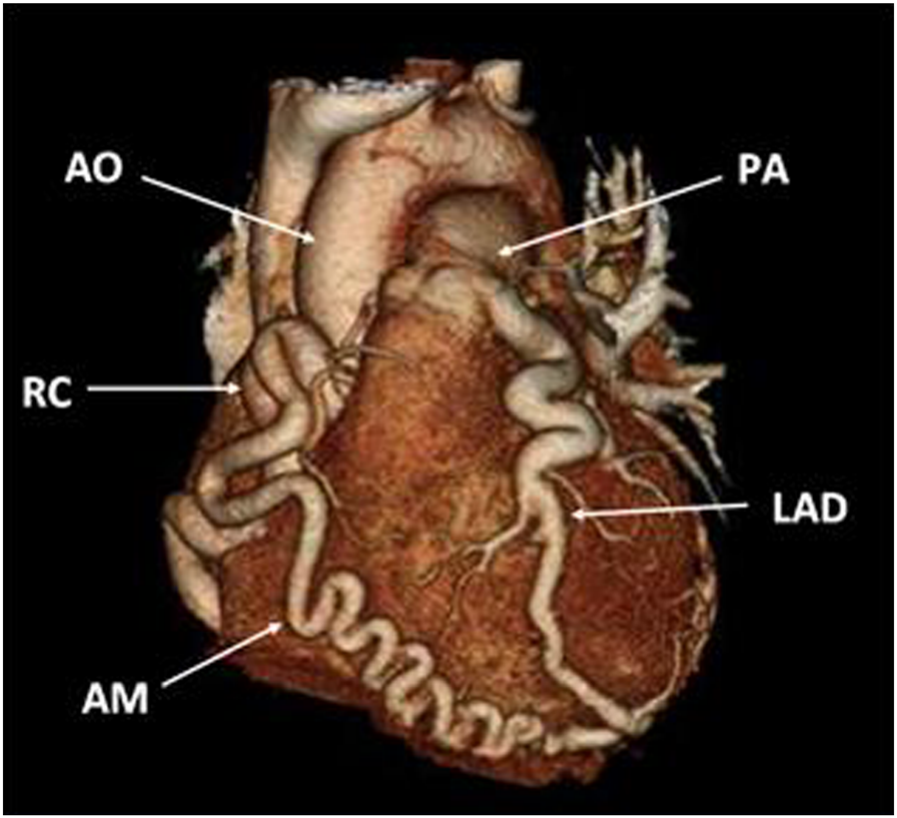
**CTA 3D reconstruction of the heart. **Aorta (AO), Right coronary artery (RC), acute marginal (AM), pulmonary artery (PA) and left anterior descending artery (LAD).

An otherwise healthy 44-year-old man presented with dyspnea on exertion and slight chest pain in 2007. Coronary angiography led to the diagnosis of ALCAPA, with a nuclear stress imaging test positive for ischemia. He initially refused surgery because the symptoms were well tolerated. Four years later, at the age of 48, the patient experienced aggravating dyspnea on exertion. Pre-operative coronary angiography showed no other anomaly than the ALCAPA. Left ventricular ejection fraction was normal on echocardiography. Cardiac computed tomography angiography (CTA) with 3D reconstruction (Figure 1) demonstrated diffusely enlarged and tortuous coronary arteries, a normal RCA implantation into the right coronary aortic sinus and a left main coronary artery (LMCA) implantation site on the pulmonary artery (PA) which was anterior and to the left [see Additional file 1]. The patient was taken to the operating room on an elective basis. Through a midline sternotomy, the position of the LMCA was confirmed, rendering anatomic re-implantation impossible. To assist in mobilizing the enlarged heart and provide hemodynamic support, cardiopulmonary bypass (CPB) was initiated in a usual fashion. Despite numerous attempts with antegrade and retrograde administration of cold blood cardioplegia, cardiac arrest was never completely achieved. After mobilization of the proximal coronary vessels, the LMCA was doubly ligated at its origin on the PA without signs of ischemia, but tissues were fragile and we found numerous small epicardial collaterals.

**Figure 2:**
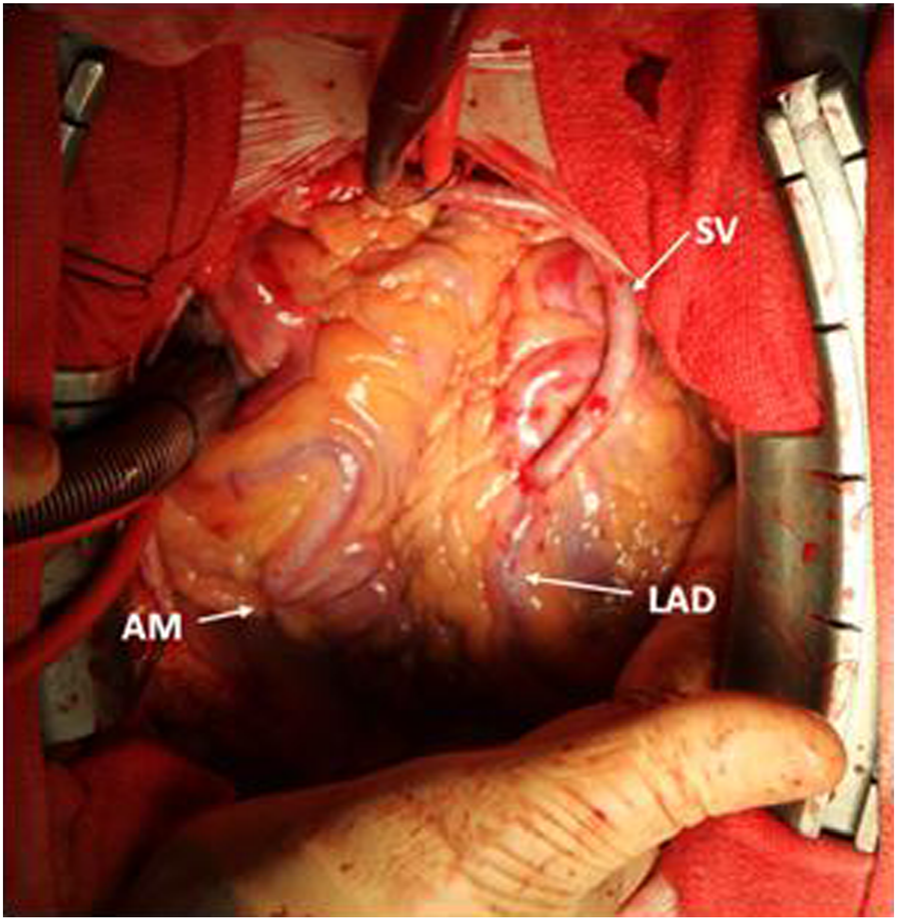
**CABG Coronary artery bypass grafting (CABG) of the left anterior descending artery (LAD) with a saphenous vein (SV) graft.** Acute marginal (AM). Patient’s head up.

In order to provide dual coronary perfusion, we proceeded with on-pump beating-heart coronary artery bypass grafting (CABG) of the left anterior descending coronary using a large saphenous vein graft (SVG) harvested in the proximal calf, selected to match the size and the significant flow in the LAD (Figure 2 and Additional file 1). Using the MediStim VeriQ flow meter, intra-operative SVG flow was measured at 117 ml/min with an excellent pulsatility index and diastolic filling. The patient was easily weaned off CPB and transferred to the intensive care unit. Post-operative course was uneventful, the patient was extubated on the same evening and he was discharged 6 days post-operatively with dual antiplatelet regimen.

## Conclusions

This exceptional case of a late presentation of ALCAPA was not only interesting from a clinical and imaging perspective but also from a therapeutic standpoint. Since patients with this anomaly rarely survive past infancy without surgical correction, very few adult patients have been reported in the literature, especially after 40 years old. We believe that patients can survive through adulthood with this anomaly without significant myocardial damage because of the development of an important collateral flow from the RCA.

Multiple procedures have been proposed in adults with ALCAPA including ligation of the left main coronary artery (LMCA), re-implantation of the LMCA to the aorta, creation of a baffle through the pulmonary artery (Takeuchi procedure) and a combination of LMCA ligation and CABG. Although re-implantation of the LMCA to the aorta remains the most physiological correction for this anomaly, the combination of LMCA ligation and CABG provides a dual coronary flow system and is preferable when re-implantation is impossible [2,3].

In this case, re-implantation of the LMCA into the aorta was considered unfeasible because of the distance between the insertion site of the LMCA on the PA and the aorta. The Takeuchi procedure was also considered but discarded because of the reported increased risk of supra-valvular stenosis as the distance increases between the insertion site of the LMCA and the junction between the aorta and the pulmonary artery [4]. End-to-end anastomosis of the LMCA with the aorta using an interposition arterial graft was not achieved because of the frailty of the surrounding tissues, the numerous collateral branches, and the inability to mobilize the bypassed vessel sufficiently to achieve an hemodynamically favorable end-to-end anastomosis. Creation of a tube graft using pulmonary artery wall autograft, as described by Wu et al. [5], with a remote LMCA insertion site in regards to the aorta, could have been another valuable option.

The collateral flow in this patient was impressive. Following LMCA ligation on CPB, cardiac arrest was not attained despite adequate cool antegrade and retrograde cardioplegia flow and relative hypothermia. In order to match the size of the large LAD, allow unrestricted flow and avoid competitive flow between the graft and the collaterals from the RCA, the largest possible conduit was selected. The choice of a large SVG conduit over an arterial graft (such as the internal thoracic or the radial artery) was in response to this situation.

In a series of 6 adult patients who underwent saphenous vein bypass grafting and direct ALCAPA closure from inside the PA, Moodie and associates reported a graft patency rate of 80% at a mean follow-up of 5.8 years [6]. Furthermore, 10-year patency of a SVG was found to be 88% when grafted to a large size LAD (larger than 2.0 mm), as reported by Goldman et al. [7]. Moreover, we obtained a SVG flow of 117 ml/min as measured at the time of surgery, which is significantly higher than what we routinely measure in LITA pedicles grafted to the LAD. We believe that this high blood flow in the SVG, along with dual antiplatelet treatment (AAS and Clopidogrel) will result in excellent long-term patency.

## Consent

Written informed consent was obtained from the patient for publication of this Case report and any accompanying images. A copy of the written consent is available for review by the Editor-in-Chief of this journal.

## Abbreviations

ALCAPA: Anomalous origin of left coronary artery from the pulmonary artery; CABG: Coronary artery bypass graft; CTA: Cardiac computed tomography angiography; LMCA: Left main coronary artery; PA: Pulmonary artery; RCA: Right coronary artery.

## Competing interests

The author(s) declare that they have no competing interests.

## Authors’ contributions

POD: Has made substantial contributions to conception and design and has been involved in drafting the manuscript. NP: Has been involved in drafting the manuscript and revising it critically for important intellectual content. JF: Has made substantial contributions to conception. LMS: Has revised the article critically for important intellectual content. CC-L: Has made substantial contributions to analysis and interpretation of data. SM: Has made substantial contributions to conception. NN: Has performed the surgery, revised the article critically for important intellectual content, is the corresponding author. All authors read and approved the final manuscript.

## Supplementary Material

Additional file 1 **Video 1.mov. **Intra-operative video showing the increase in size of the tortuous coronaries on the beating heart. Click here for file
